# Autism as a Disorder of Biological and Behavioral Rhythms: Toward New Therapeutic Perspectives

**DOI:** 10.3389/fped.2015.00001

**Published:** 2015-02-23

**Authors:** Sylvie Tordjman, Katherine S. Davlantis, Nicolas Georgieff, Marie-Maude Geoffray, Mario Speranza, George M. Anderson, Jean Xavier, Michel Botbol, Cécile Oriol, Eric Bellissant, Julie Vernay-Leconte, Claire Fougerou, Anne Hespel, Aude Tavenard, David Cohen, Solenn Kermarrec, Nathalie Coulon, Olivier Bonnot, Geraldine Dawson

**Affiliations:** ^1^Laboratoire Psychologie de la Perception, Université Paris Descartes, CNRS, UMR 8158, Paris, France; ^2^Pôle Hospitalo-Universitaire de Psychiatrie de l’Enfant et de l’Adolescent (PHUPEA), Centre Hospitalier Guillaume Régnier, Université de Rennes 1, Rennes, France; ^3^Department of Psychiatry and Behavioral Sciences, Duke University School of Medicine, Durham, NC, USA; ^4^Department of Child and Adolescent Psychiatry, Centre Hospitalier Le Vinatier, Lyon, France; ^5^Department of Child and Adolescent Psychiatry, Centre Hospitalier de Versailles, Université de Versailles Saint-Quentin-en-Yvelines, Le Chensay, France; ^6^Child Study Center, Yale University School of Medicine, New Haven, CT, USA; ^7^Department of Child and Adolescent Psychiatry, Assistance Publique – Hôpitaux de Paris, Groupe Hospitalier Pitié-Salpêtrière, CNRS FRE 2987, University Pierre and Marie Curie, Paris, France; ^8^Service Hospitalo-Universitaire de Psychiatrie de l’Enfant et de l’Adolescent, CHU de Brest, Université de Bretagne Occidentale, Brest, France; ^9^Clinical Investigation Center, INSERM CIC 0203, University Hospital, Rennes 1 University, Rennes, France; ^10^Department of Clinical Pharmacology, University Hospital, Rennes 1 University, Rennes, France; ^11^Pôle Hospitalo-Universitaire de Psychiatrie Adulte (PHUPA), Centre Hospitalier Guillaume Régnier, Université de Rennes 1, Rennes, France

**Keywords:** autism spectrum disorder, biological rhythms, motor, emotional and relational rhythms, synchronization of rhythms, melatonin, Early Start Denver Model, therapeutics

## Abstract

There is a growing interest in the role of biological and behavioral rhythms in typical and atypical development. Recent studies in cognitive and developmental psychology have highlighted the importance of rhythmicity and synchrony of motor, emotional, and interpersonal rhythms in early development of social communication. The synchronization of rhythms allows tuning and adaptation to the external environment. The role of melatonin in the ontogenetic establishment of circadian rhythms and the synchronization of the circadian clocks network suggests that this hormone might be also involved in the synchrony of motor, emotional, and interpersonal rhythms. Autism provides a challenging model of physiological and behavioral rhythm disturbances and their possible effects on the development of social communication impairments and repetitive behaviors and interests. This article situates autism as a disorder of biological and behavioral rhythms and reviews the recent literature on the role of rhythmicity and synchrony of rhythms in child development. Finally, the hypothesis is developed that an integrated approach focusing on biological, motor, emotional, and interpersonal rhythms may open interesting therapeutic perspectives for children with autism. More specifically, promising avenues are discussed for potential therapeutic benefits in autism spectrum disorder of melatonin combined with developmental behavioral interventions that emphasize synchrony, such as the Early Start Denver Model.

## Introduction

Endogenous physiological variations involved in biological rhythms reflect adaptation to the environment. Thus, the sleep–wake rhythm associated with biological circadian rhythms can be viewed as an adaptation to the day–night cycle. Circadian rhythms allow temporal organization of biological functions in relation to environmental changes (1). The periodicity of activities applies to all biological, physiological, and psychological functions; recently, the science of biological rhythms, chronobiology, has emerged with its own theory, science, and education (2).

Furthermore, recent studies in the field of cognitive and developmental psychology have highlighted the importance of rhythmicity and synchrony of motor, emotional, and relational rhythms in early development of social communication. Given the major role of the sleep hormone melatonin in the ontogenetic establishment of diurnal rhythms, the synchronization of peripheral oscillators (also termed clocks) and the regulation of human circadian rhythms (1), melatonin might be involved in the synchrony of motor, emotional, and relational rhythms.

Indeed, relationships might exist, based on the hypothesis of ergodicity (3), between cellular communication networks involving a cellular synchrony (synchronization of cellular oscillations by melatonin) and early social communication development involving a synchrony of motor, emotional, and interpersonal rhythms. Autism spectrum disorder (ASD) – a developmental disorder characterized by social communication impairments associated with repetitive interests and behaviors – provides an interesting and challenging model of abnormal melatonin production in early developmental disorders and its possible relationship with autistic behavioral impairments.

This article proposes a central role of rhythmicity and synchrony of rhythms in typical child development and offers a new integrative approach, which considers autism as a disorder of biological and behavioral rhythms. In this perspective, promising avenues will be discussed in this article for potential therapeutic benefits in ASD of melatonin and developmental behavioral interventions that emphasize rhythms and synchronization, such as the Early Start Denver Model (ESDM).

## Physiological and Behavioral Rhythm Disturbances in Autism

### Autism as a disorder of biological rhythms

Alterations in circadian sleep–wake rhythm have frequently been reported in autism (4, 5). More specifically, reduced total sleep and longer sleep latency as well as nocturnal and early morning awakenings are often observed in individuals with ASD (6–12). Furthermore, prior studies on melatonin in autism have all reported abnormalities in melatonin secretion (see Table 1). In addition, abnormalities in cortisol circadian rhythm have also been reported in autism [for a review, see Tordjman et al. (13)]. In particular, significantly higher frequency of absence of circadian variation in melatonin and cortisol levels was observed in individuals with autism compared to typically developing controls. Golombek et al. (14) described the effects of circadian desynchronization that can enhance susceptibility to certain disorders (metabolic, immune, cognitive, and somatic disorders including cancer). It is noteworthy that congenitally blind children with consequently abnormal melatonin secretion and synchronization [the production of pineal-derived melatonin depends on the light acting through the retinohypothalamic tract (15)] very frequently display autism [up to 42% (16)], whereas hearing impaired children, including hearing loss, show autism less frequently [up to 10% (17)]. More specifically, abnormally low daytime and nighttime melatonin secretion was associated with an absence of melatonin circadian variation in some individuals with autism (18, 19), which in turn, given the role of synchronizer of melatonin, also has consequences on the circadian rhythms network, including the cortisol circadian rhythm (13, 20).

**Table 1:** **Studies of melatonin levels in individuals with autism**.

Study	Sample	Study group	Measured variable	Results
Ritvo et al. (21)	Urine	Young adults with autism (*N* = 10)	Melatonin concentration	Increased daytime values compared to typically developing controls
				Similar nighttime values compared to typically developing controls
Nir et al. (22)	Serum	Young men with autism (*N* = 10)	Melatonin concentration	Increased daytime values compared to typically developing controls
				Decreased nighttime values compared to typically developing controls
Kulman et al. (19)	Serum	Children with autism (*N* = 14)	Melatonin concentration (24-h circadian rhythm)	Decreased nighttime values compared to typically developing controls
				No circadian variation in 10/14 (71.4%) children with autism
				Inverted rhythm in 4/14 (28.6%) children with autism
Tordjman et al. (23)	Urine	Children and adolescents with autism (*N* = 49)	6-Sulphatoxymelatonin excretion rate (12-h collection)	Decreased nighttime values compared to typically developing controls
Melke et al. (24)	Plasma	Adolescents and young adults with autism (*N* = 43)	Melatonin concentration	Decreased daytime values compared to typically developing controls
Mulder et al. (25)	Urine	Children and adolescents with autism (*N* = 20)	6-Sulphatoxymelatonin excretion rate (24-h collection)	Trend to lower 24-h melatonin excretion rate in hyperserotonemic compared to normoserotonemic individuals with autism
Tordjman et al. (18)	Urine	Post-pubertal adolescents and young adults with autism (*N* = 43)	6-Sulphatoxymelatonin excretion rate (split 24-h collection)	Decreased daytime values compared to typically developing controls
				Decreased nighttime values compared to typically developing controls
				No circadian variation in 10/43 (23.2%) individuals with autism

This blunted circadian rhythmicity with no or little variability might be related to the difficulties in adapting to changes typically observed in individuals with autism. Thus, children with autism who are confronted with physiological continuity due to absent circadian rhythms may have difficulties adapting to changes in either their external or their internal environment (26). Indeed, as previously underlined, the circadian clocks network, synchronized by melatonin and involving an internal system of continuity/discontinuity, allows adaptation to environmental changes.

Similarly, blunted circadian rhythmicity may explain the difficulty observed in many children with autism in adapting to changes in rhythms of their external and internal environment. Thus, rapid rhythms of sensory stimuli in their external environment (e.g., repeated visual stimuli provided by a stroboscopic light) can provoke epileptic seizure in some individuals with autism [approximately one-third of children with autism have epilepsy (27)]. Interestingly, EEG changes tended to be correlated with an abnormal rhythm of melatonin in young adults with autism (22). Furthermore, some parents have reported that their daughters with autism experience epileptic seizures toward the 14th day of their menstrual cycle, which is when luteinizing hormone (LH) levels peak (Tordjman, personal communication), suggesting quite tentatively that individuals with autism may have difficulties adapting to periodic hormonal changes in their internal environment. We can state the following hypothesis: a change in rhythm associated with excessive environmental stimuli might strongly increase arousal and lead to physiological stress which, for some individuals with autism, can disturb the rhythmic activity of a particular brain area, leading it to fall out of sync with the rest of the brain and causing its population of neurons to fire (depolarization and occurrence of an epileptic seizure). This underlines the importance of stable physiological rhythms.

Finally, significant relationships have been found between lower nocturnal melatonin excretion and increased severity of autistic social communication impairments, especially for verbal communication and social imitative play (18, 23). These findings are in agreement with studies suggesting an association between reduced melatonin production and language impairment (22, 28). Along the same line, the systemic administration in the animal model of Zebra Finch of a melatonin-1B receptor antagonist at the beginning of the night shortens the song and motif length and affects the song syllable lengths produced the next day (29). Reduced melatonin activity might create timing problems in biological clocks with physiological and psychological effects that might be, according to Boucher’s model of autism (30) and Wimpory’s theory (31), involved in autistic impairments, notably in autistic social communication impairments. It is noteworthy that deficiency in oxytocin [oxytocin is considered a bonding hormone (32, 33)] has also been reported in autism (34, 35) and bonding is involved in the development of very early social interaction in infants. Interestingly, the release of oxytocin by the posterior pituitary gland follows a robust circadian rhythm in mammals. Further studies are needed to better understand the underlying mechanisms of oxytocin anomalies in autism and to explore, in particular, possible oxytocin rhythm disturbances in ASD. The importance of the synchrony of rhythms in the development of social interaction and communication is detailed in the next section.

### Importance of synchrony of rhythms for social communication in typical development and autism spectrum disorder

Several studies, based on animal models and human perinatal development, suggest that stable patterns of repeated stimuli in the form of maternal physiological rhythms, involving cross-modal perception such as regular cardiac rhythm, which provides the fetus with auditory and vibratory stimuli, allow the fetus to integrate sensory information facilitating prenatal perceptual learning and develop a coherent representation of his or her internal and external environment (36–38). Fluctuations in the physiological rhythms (variants), such as variations in the maternal cardiac rhythm and also variations in hormone levels involved in the circadian rhythms that are already present during the fetus life (the fetus’ circadian rhythms are the mother’s ones), occurring in a background of regular repetition of identical sequences (invariants), may help the fetus to develop the ability to adapt to change in an environment characterized by high regularity. As previously emphasized (38), very early mother–infant relations provide a secure environment based on the repetition of invariants, while at the same time promoting adaptation to change through the presence of variants. It is through the regular repetition of identical sequences of discontinuity, such as circadian rhythms, that a continuum is constructed associated with the development of adaptation to changes.

The development of the very earliest form of communication relies on the sharing of emotions between mother and infant, when, for example, the infant is suckling in his or her mother’s arms, through emotional synchrony that enhances the integration of sensory inputs (39, 40). Concerning memory processes, emotions enable to “fix” events, just as a photographic fixing agent sets images (38). Cortisol (a stress and arousal neurohormone) crosses the placental barrier. The cortisol circadian rhythm, as well as the melatonin circadian rhythm in the fetus and infant after birth, are those of maternal cortisol and melatonin. Indeed, the infant’s circadian cortisol and melatonin rhythms are only established between 2 and 3 months of age in typical development, at the same time that infants begin to have more regular sleep–wake cycles associated with nighttime sleep lasting 6–8 h (41, 42). Interestingly, this period coincides in typical development with the emergence of social smiling by the second month of life (43), the advent of mirror self-recognition at around 3 months of age (44), and increased brain activation to speech occurring between 3 and 4 months of age (45).

At birth, the human immaturity of the cerebral cortex allows initial learning to influence the neural architecture through perceptual-action mapping (46, 47). The infant’s social skills, especially including imitation, shared attention, and empathic understanding, also contribute to the development of learning (46, 48). Social synchrony can be defined as the dynamic and reciprocal adaptation of the temporal structure of behaviors between interactive partners (49). In typically developing children, the quality of social interaction depends on an active dialog between the parent and the infant (50, 51). Numerous studies have been emphasizing the importance of parent–infant synchrony and the construction of shared timing in social communication development (52).

Also, biological markers were associated with relational synchrony. First, oxytocin administration to parent enhances infant physiological and behavioral readiness for social engagement and parallels an oxytocin increase in infants (32). Second, neural correlates were found using hyper-scanning recordings of EEG brain activity and measures of neural synchronization between distant brain regions of interacting individuals through a free imitation task (53). Dumas and colleagues’ study (53) was the first to record dual EEG activity in dyads of subjects during spontaneous non-verbal interaction. Eleven same-sex pairs were scanned. They found that interpersonal rhythmic oscillations were correlated with the emergence of synchronization in the brain’s alpha–mu band between the right centro-parietal regions (an area involved in social interaction) of both participants. Developmental psychologists now study interaction not only as the addition of two behaviors but also as a global phenomenon in which synchrony is considered as social *per se*. To describe the dialog between two partners engaged in behavioral and affective exchange, developmental psychologists more and more take into consideration rhythm and temporal course of both behavior and affect, regarded as key expressions of adaptation during interaction (49, 52).

Only a few studies have addressed the importance of infant–caregiver synchrony/reciprocity in the development of social communication involving infants who subsequently are diagnosed with autism. It appears appropriate in the field of autism to consider the combined domain of social communication, as the most recent version of the ADOS scale does, as well as the recently released DSM-5 American classification. Methods to investigate this issue include studies using early home videos (54), parental interviews focusing on early abnormalities (55), and prospective assessment of children at risk of ASD (e.g., siblings) (56, 57). Studies have revealed a pervasive developmental course in infants who were later diagnosed with ASD. Thus, the first signs were abnormalities in eye contact, imitation, disengagement, joint attention, orienting to name, and body language. These behaviors are parts of the affective tuning disturbances; the term affective tuning, defined by Stern (58) as the “execution of behaviors expressing the emotional ownership of a shared affective state,” refers to infant–caregiver emotional communication based on rhythmic similarity from the second semester of life onward. Also, these behaviors are important precursors of later-developing symptoms. However, whether these first signs impair early infant–parent interactions and whether they reflect already autistic behavioral impairments in the infant remain to be explored. In two related studies based on home movies of children later diagnosed with autism, Saint-Georges et al. (54) and Cohen et al. (59) showed that motor and emotional asynchrony was present between infants and parents before 12 months of age, and parents perceived weaker initiatives from their children. In addition, parents experienced weaker interactive responsiveness from their children and increasingly tried to compensate this perceived deficit by soliciting behaviors through touching the infant. This was particularly observed after 12 months of age for the fathers of infants who were later diagnosed with autism.

Many authors have studied imitation in children with autism (imitation of other people’s faces, gestures, or vocal signals) in order to better understand the development of autistic social communication impairments. This specific type of imitation is referred to as “spatial” imitation to highlight the capacity to produce an instantaneous copy of the form of the signal. However, another way to communicate with others is to perform a “temporal” imitation of their behavior (60). This is what humans do through rhythmic finger or foot taping, dancing, singing, and drumming in synchrony with others (61). Xavier et al. (62) highlighted the importance of rhythmicity and synchrony in the development of children’s imitative exchanges with peers. From birth, a child has a predisposition to engage, intersubjectively, with the rhytmic actions and awareness of other persons, and to move in synchrony with them (44, 63). Synchronic imitation is an important preverbal way to communicate among peers (64, 65). This reciprocal experience concerns two children able and motivated to coordinate their behavior with the non-ritualized behavior of the other, in both form and timing and to alternate turns between model and imitator (66). The impression of fluidity in the coordination of movements between partners is underlined by mutual attention, engagement, continuous adaptation, and turn taking (67). This rhythmic process made of ludic spontaneous imitation reveals moments of discontinuity occurring in a background of continuity. Neural bases of this coupling activity are constituted by the neuron mirrors system (68), with the same evidence showing that the neuronal structures involved when a mental state is experienced, are also recruited during the observation of others. Guionnet et al. (69) designed a free imitation paradigm in an fMRI study to examine some neural correlates of social interaction. Their results agree with those previously evidenced (70, 71) concerning the core circuit of imitation, but they found different activations between the situation of imitating and that of being imitated.

A special quality of temporal imitation is the ability to use different motor movements in order to communicate. Thus, simple finger tapping can be synchronized with another’s head nodding or trunk movements, whether entrained by one of the movements or in response to external synchronizing stimuli such as music. Although animals and humans can perceive rhythms and produce rhythmic motor patterns, only humans can adapt their rhythmic movements to external rhythms (72) [with the exception of the cockatoo (73)]. The ability to be rhythmically synchronized with the environment appears important for infant development in the emotional, cognitive, social, and sensorimotor realms (44, 74). It has been demonstrated that the human fetus and newborn already have the capacity to perceive and produce rhythms (75). The ability to produce temporally adapted motor patterns comes later and depends on the specific motor system involved and the relationship between the beat presented and the spontaneously occurring motor tempo of the infant (76, 77). It should be fruitful to longitudinally examine children with autism in terms of ability to adapt their own rhythm to external rhythms. Interestingly, clinical observations suggest that some children with autism are able to respond to an external rhythmic vocalization by a similar rhythmic motor pattern such as hand flapping (Tordjman, personal communication). However, previous studies reported disorganized rhythms, stereotypies, and poor synchrony in most of these children (78), which might be related to the low melatonin levels reported to be associated in autism with the severity of verbal communication and social imitative play impairments (18, 23). Melatonin, as a regulator of physiological rhythms and oscillations, might enhance the capacity of children with ASD to synchronize their movements with movements of others (this synchronization of movements is needed for imitative play) and with external rhythmic auditory stimuli (such as music and/or human voice enhancing their verbal skills). Interestingly, in a study of social smiling in infants (79), there was no difference in frequency of smiling between 2- and 5-month-old infants with and without ASD during infant–caregiver face-to-face interactions. However, whereas typically developing infants showed a significant increase in smiling rate when caregivers were smiling, smiling in infants later diagnosed with ASD was not synchronized with smiling in caregivers and was not contingent upon caregiver behavior (caregiver facial expressions and vocalizations). Furthermore, it is noteworthy that, as previously indicated in this section, the infant’s melatonin circadian rhythm is established between 2 and 3 months of age, and a study (80) reported that eye contact was normal in 2-month-old infants later diagnosed with autism but declined between 2 and 6 months of age, suggesting an additional argument in favor of a possible relationship between the well-replicated nocturnal melatonin deficit in autism (see Table 1) and the development of autistic social communication impairments.

### Repetitive behaviors and interests

Repetitive behaviors and interests are defined, according to DSM-5 criteria (81), as a repetition of identical sequences of behaviors for motor stereotypies (motor stereotypies involve repetitive maladaptive movements) or thoughts for restrictive patterns of interests (restrictive and repetitive interests involve fixated interests, adherence to routines, or ritualized and rigid thinking patterns). Thus, repetitive behaviors and interests can be viewed as behavioral responses to the need to create discontinuity that is repeated at regular intervals, which could have been fundamentally lacking in the physiological development of children with autism due to the melatonin deficit reported in autism. Our finding (18) observed in a sample of 43 adolescents and young adults with autism (nocturnal excretion of 6-SM was significantly negatively correlated with repetitive use of objects), taken together with improvement of stereotyped behaviors following administration of melatonin in 24 children and adolescents with ASD (82), supports this hypothesis.

The autistic deficit in melatonin secretion might lead to physiological rhythm disturbances in autism impairing biological circadian rhythms and even, in certain cases, to an “endless” physiological continuity provoked by the absence of variation in melatonin levels. From this perspective, stereotyped behaviors and interests can be seen as offering to children with autism rhythmic forms providing rhythmicity and discontinuity through the creation of repeated identical patterns. Albert Goldbeter (83), director of the Chronobiology Unit at the Brussels Sciences University, underlines that life is rhythm. It is noteworthy that the word pineal comes from pine cone that is a Mesopotamian symbol representing the source of life and the power of regeneration. In Asia, the pineal gland is considered as the 7th chakra and represents the location of the soul and the highest level of consciousness.

Donald Winnicott (84) emphasized that “the main problem for a typically developing child is to be able to create a continuum out of discontinuity.” According to him, the first optimal container (“holding environment”) for the newborn is the progressive internalization of the rhythmic structures of feeding (rhythmic ebb and flow corresponding to the kinesthetic experience of suckling), providing a sense of continuing existence. We can nevertheless hypothesize, as we did in the previous section and in a prior article (26), that this internalization process starts far earlier, in the womb, through maternal physiological rhythms; conversely, based on clinical observations in autism, children with autism create discontinuity out of continuity. We can also state the hypothesis that children with autism, confronted with an “endless” physiological continuity (or at least with an absence of physiological discontinuities repeated at regular intervals), would develop anxiety and stress due to the lack of secure stable rhythms, and would therefore try to create by themselves rhythmicity in order to control them through stereotyped behaviors. However, the relationships between stereotyped behaviors and stress responses or anxiety need to be clarified given discrepant results reported in some studies (85, 86).

Finally, it should be noted that several studies have also recently opened new behavioral, neurobiological, and pharmacological perspectives on autistic repetitive behaviors, especially on self-injurious behaviors [for a review, see Adler et al. (87), Minshawi et al. (88), and Stigler (89)]. Thus, research on potential therapeutic effects for repetitive behaviors and interests should investigate benefits of melatonin in ASD (see next section) but should not be limited to the administration of melatonin.

## Therapeutic Benefits of Melatonin in Autism Spectrum Disorder

Published melatonin treatment studies in autism are presented in Table 2. Many have been most concerned with effects of melatonin on sleep. Reviews, commentaries, and meta analyses of melatonin treatment studies are presented in Table 3. These two tables come from a prior article of our team (26).

**Table 2:** **Studies on potential therapeutic benefits of melatonin in autism**.

Study	Journal	Population	Design	Duration of treatment	Melatonin (formulation, dose)	Time of intake	Main outcome measures	Effects on sleep	Other outcomes	Comments
**SINGLE CASE REPORTS**										
Horrigan and Barnhill (90)	*J Am Acad Child Adolesc Psychiatry*	17-year-old boy with Asperger’s Syndrome (AS)	–	Not given	3 mg	20–30 min before bedtime (BB)	Sleep	Sleep improvement. No side effects	Daytime behavior improvement	–

Hayashi (91)	*Psychiatry Clin Neurosci*	14-year-old boy with autistic disorder, severe intellectual disability and phase delay with polyphasic sleep	–	4 months	Immediate release (IR) 6 mg	11:00 p.m.	Sleep	Melatonin increased sleep duration. No side effects	None	–

Jan et al. (92)	*Dev Med Child Neurol*	12-year-old boy with AS and complex sleep disturbance (phase delay and parasomnias)	–	6 months	Controlled release (CR) 5 mg	30 min BB	Sleep	Normalization of the sleep–wake rhythm and disappearance of parasomnias. No side effects	None	–
**RETROSPECTIVE STUDIES**										
Gupta and Hutchins (93)	*Arch Dis Child*	9 cases of children with autistic disorder (AD) aged from 2 to 11 years. Chronic sleep problems	Not given	1 week to 1 year	IR 2.5–5 mg	45 min BB	Parental evaluation of sleep	56% showed improvement in total sleep duration	None	No standardized collection of sleep variables

Andersen et al. (94)	*J Child Neurol*	107 children and adolescents aged from 2 to 18 years with ASD (DSM-IV): 71% AD, 5% AS, 19% PDDNOS (pervasive developmental disorder not otherwise specified)	Not given	Mean duration: 1.8 years	IR in 91% of the cases. Dose escalation protocol from 1 to 6 mg based upon age	30–60 min BB	Parental evaluation of sleep	Parents reported full (25%) or partial (60%) improvement. Beneficial effects of melatonin seem to stop after 3–12 months despite the use of higher doses. Side effects observed in 3 children: sleepiness, fogginess, increased enuresis	None	No standardized collection of sleep variables. The loss of response to melatonin treatment is discussed in the text

Galli-Carminatti et al. (95)	*Swiss Med Wkly*	6 adult patients with AD (CIM-10) and intellectual disability, aged from 19 to 52 years	Not given	6 months	IR. Dose escalation protocol from 3 to 9 mg if clinically required	45 min BB	Sleep (CGI-S and CGI-I)	Improvement in sleep onset latency, night and early morning awakenings. No side effects	None	No standardized collection of sleep variables. Two to four associated psychotropic drugs per patient
**OPEN-LABEL TRIALS**										
Jan et al. (96)	*Dev Med Child Neurol*	15 children with multiple neurological disabilities and severe sleep disorders	Not given	Not given	2–10 mg	Bedtime	Not given	Partial improvement in sleep disorders. No side effects	Behavior and social improvement	Heterogeneous sleep disorders and neurological disabilities

Ishizaki et al. (97)	*No To Hattatsu*	50 children and young adults with autism (*n* = 27) or mental retardation (*n* = 20) or severe motor/intellectual disability (*n* = 3) aged from 3 to 28 years with sleep disorders	Not given	Not given	Not given	Not given	Sleep disorders and emotional/behavior disturbances	34 patients experienced improvement in response to melatonin. Side effects reported in 17 patients	Improvements in excitability when sleep also improved. No change in contrariness, stereotyped behavior and in school/workshop refusal	Various types of insomnia and diagnoses

Paavonen et al. (98)	*J Child Adolesc Psychopharmacol*	15 children with AS (DSM-IV) aged from 6 to 17 years with severe sleep problems for at least 3 months	Not given	14 days	IR 3 mg	30 min BB	Sleep (72 h-period actigraphy, sleep diaries), daytime behavior (Karolina Sleepiness Scale; KSS), Child Behavior Checklist (CBCL)	Melatonin treatment was associated with significant decrease in sleep onset latency and nocturnal activity. Discontinuation of melatonin led to a significant decrease in sleep duration and more nocturnal activity. Side effects in 20% of the cases: tiredness, headaches, severe sleepiness, dizziness, diarrhea	Significant improvement of daytime behavior (CBCL)	No principal outcome specified. KSS is not validated in children nor in ASD

Giannotti et al. (99)	*J Autism Dev Disord*	29 children with AD (DSM-IV) aged from 2 to 9 years with current sleep problems	Controlled-release melatonin	6 months	Dose escalation protocol from 3 mg (1 mg of IR + 2 mg of CR) to 6 mg when clinically required, based upon age (max 4 mg under 4 years old and max 6 mg over 6 years old)	08:00 p.m.	Sleep (diaries and Children’s Sleep Habits Questionnaire CSHQ), daytime behavior, Childhood Autism Rating Scale (CARS)	Melatonin treatment was associated with improvement in sleep onset latency, night awakenings, and sleep duration, which vanished after melatonin discontinuation. No side effects	Parents reported less irritability, less anxiety, and better mood. Significant improvement of depression, anxiety, and withdrawal symptoms during melatonin treatment in children with AS. No effect was reported on the CARS	No principal outcome specified. Missing data: analyses on 25 patients

De Leersnyder et al. (100)	*Pediatr Neurol*	88 children with heterogeneous neurodevelopmental disorders (Smith-Magenis syndrome, mental retardation, encephalopathy, Angelman syndrome, Rett syndrome, Bourneville syndrome, blindness, and autism) aged from 5 to 20 years. Seven patients with autism, mean age 12 years old	6 years of open-label follow up	3 months	CR 2–4 mg (<40 kg) or 6 mg (>40 kg) based upon weight	60 min BB	Parental evaluation of sleep and mood (self-constructed questionnaire)	According to parental reports, both sleep latency and sleep duration improved within 3 months such as night awakenings, sleep quality, and daytime napping. Eleven children experienced adverse events (daytime nap, difficulties in swallowing tablets) that the parents attributed to melatonin treatment	12% of the parents reported improvements of mood in their children	Heterogeneous neurodevelopmental disorders. Results cannot apply to a population with autism spectrum disorders. No standardized collection of sleep and mood parameters. Mean dose for patients with autism: 5.7 mg

Malow et al. (82)	*J Autism Dev Disord*	24 children with ASD (DSM-IV, ADOS): AD, AS, and PDDNOS aged from 3 to 9 years. Sleep onset delay of 30 min or longer confirmed on actigraphy. Exclusion of neurodevelopmental disabilities such as fragile X, Down, and Rett syndromes	Before treatment families received structured sleep education and children underwent a treatment acclimatation phase in order to be sure the melatonin will be taken	14 weeks	CR. Dose escalation protocol from 2 to 9 mg when clinically required	30 min BB	Sleep (actigraphy, Children’s Sleep Habits Questionnaire; CSHQ, diaries), daytime behavior (Child Behavior Checklist; CBCL, Repetitive Behavior Scale-Revised), parental stress (Parenting Stress Index Short Form), side effects (Hague Side Effects Scale)	Significant improvement in sleep latency within the first week of treatment but not for other sleep parameters such as night awakenings and sleep quality	Significant improvement in children’s behavior (withdrawal, affective problems, attention-deficit hyperactivity, stereotyped, and compulsive behaviors). Significant improvement in parental stress	No placebo
**PLACEBO-CONTROLLED TRIAL**										
McArthur and Budden (101)	*Dev Med Child Neurol*	9 children and adolescents with Rett syndrome aged from 4 to 17 years. Mean age:10 years old	Randomized double-blind crossover trial	2 periods of 4 weeks with a wash out period of 1 week	2.5–7.5 mg based on weight	60 min BB	Sleep (actigraphy, diaries)	Significant improvement in total sleep time. No side effects	None	–

Garstang and Wallis (102)	*Child Care Health Dev*	11 children and adolescents with ASD aged from 5 to 15 years with chronic sleep disorders resistant to behavioral treatment	Randomized double-blind crossover trial	2 periods of 4 weeks with a wash out period of 1 week	IR 5 mg	60 min BB	Sleep (diary)	Melatonin and placebo were associated with significantly decreased sleep latency and nocturnal awakenings, increased total sleep time. No side effects	Several parents and class teachers commented that their children were easier to manage and less rigid in their behavior while taking melatonin	ASD criteria were not consensual. Only 7 children completed the trial. Investigators found that some of the placebo capsules were empty. Missing data

Wasdell et al. (103)	*J Pineal Res*	51 children and adolescents with neurodevelopmental disabilities (16 patients with ASD) aged from 2 to 18 years. Sleep delay phase syndrome and impaired sleep maintenance with resistant to sleep hygiene intervention	Randomized double-blind crossover trial. Three weeks trial followed by a 3-month open-label study. Behavioral sleep treatment before inclusion	2 periods of 10 days with a wash out period of 3–5 days	Dose escalation protocol based on unspecified conditions: from 5 mg (1 mg FR + 4 mg CR) to 15 mg	20–30 min BB	Sleep (actigraphy, diaries, CGI-S, CGI-I), familial stress (family stress scale)	Significant improvement in total sleep duration and sleep latency as well as reduced stress levels in parents in the melatonin arm	Half of the patients with ASD had their dose increased during the open-label phase with no additional improvement in sleep latency or sleep duration, but caregivers reported less anxiety	Unspecified ASD criteria. Fifty patients completed the trial and 47 completed the open-label phase. Selection bias due to previous melatonin treatment (25% of the cases). At the end of the trial, 29 patients received a dose of 10 or 15 mg. Higher doses were necessary in patients with bilateral cerebral lesions

Wirojanan et al. (104)	*J Clin Sleep Med*	12 children and adolescents with unspecified sleep problems, aged from 2 to 15 years: 5 patients with AD (ADOS and ADI-R), 3 patients with fragile X syndrome with AD, 3 patients with AD and fragile X syndrome, and 1 patient with fragile X premutation	Randomized double-blind crossover trial	2 periods of 2 weeks. No wash out period	IR 3 mg	30 min BB	Sleep (actigraphy, diary)	Significant, but mild improvement in total sleep time (+21 min) and decrease in sleep latency (−28 min)	None	Missing data: only 12 patients completed the trial (order bias). No subgroup analysis in AD patients. No side effects

Wright et al. (105)	*J Autism Dev Disord*	22 children and adolescents aged from 3 to 16 years with ASD (ICD-10, ADOS, ADI-R): AD (70%), AS (10%), and AA (20%). No fragile X or Rett syndrome. Current sleeplessness (confirmed on a 1-month-diary) and resistant to behavioral treatment	Randomized double-blind crossover trial	2 periods of 3 months separated by 1 month of washout	IR. Dose escalation protocol from 2 to 10 mg when clinically required	30–40 min BB	Sleep (sleep difficulties questionnaire, diary), daytime behavior (Developmental Behavior Checklist), side effect questionnaire	Significant improvement in sleep latency (−47 min) and total sleep duration (+52 min) in the melatonin arm. No improvement in night awakenings. The side effect profile was not significantly different between the 2 groups	Improvement in children’s behavior in the melatonin arm that was significant for communication (*p* = 0.045)	Missing data. Analysis on 16 patients. No actigraphy. Mean melatonin dose: 7 mg

Cortesi et al. (106)	*J Sleep Res*	160 children with ASD (DSM-IV, ADI-R, ADOS) aged from 4 to 10 years with sleep onset insomnia and impaired sleep maintenance	Randomized placebo-controlled. Randomization in 4 groups: (1) melatonin alone (2) melatonin+ cognitive behavioral therapy (CBT) (3) CBT alone (4) placebo	12 weeks	CR 3 mg	09:00 p.m.	Sleep (actigraphy, Children’s Sleep Habits Questionnaire, diaries)	144 patients completed the trial and 134 were analyzed. Combination group showed greater significant improvements on sleep followed by the melatonin alone and the CBT alone compared to placebo group. No side effects	None	–

Gringas et al. (107)	*BMJ*	146 children aged from 3 to 15 years with neurodevelopmental disorders (60 patients with ASD) and severe sleep disorders that did not respond to standardized sleep advice	Double-blind randomized multicentre placebo-controlled phase III trial	12 weeks	Immediate release melatonin (dose escalation protocol from 0.5 to 12 mg) or matching placebo	45 min before bedtime	Total sleep time after 12 weeks (sleep diaries and actigraphy); sleep onset latency; child behavior (Aberrant Behavior Checklist); family functioning; adverse events	Melatonin increased total sleep time by 22.4 min (diaries) and 13.3 (actigraphy); reduced sleep onset latency by 37.5 min (diaries) and 45.3 (actigraphy). Children in the melatonin group woke up earlier than the children in the placebo group. Melatonin was most effective in children with longest sleep latency. Adverse events were similar between the 2 groups	Child behavior and family functioning outcomes showed some (but not significant) improvement and favored use of melatonin	The results are not specified by category of developmental disorder

**Table 3:** **Review, meta-analysis, and discussion of therapeutic uses of melatonin in autism**.

**REVIEW/META-ANALYSIS**		
Jan and O’Donnel (108)	*J Pineal Res*	Review based on100 individuals with chronic sleep disorders, aged from 3 months to 21 years. Half of these 100 patients presented visual impairment or blindness. Melatonin dose ranged from 2.5 to 10 mg. Higher doses were needed in patients with impaired sleep maintenance. Partial or total improvement in sleep parameters was found in 82% of the cases. No side effects
Jan et al. (109)	*Dev Med Child Neurol*	Systematic review of studies on melatonin in children. Twenty-four studies found, most of them were case reports or uncontrolled studies with small samples. Mean age: 10 years old. Associated diagnosis: blindness and neurodevelopmental disabilities, 1 single case of an adolescent with AS (76). Doses ranged from 0.5 to 20 mg. Improvement in sleep in all the studies
Phillips and Appleton (110)	*Dev Med Child Neurol*	Only three studies, reporting a total of 35 children, fulfilled the criteria for inclusion (randomized controlled clinical trials). Two of them reported a significant decrease in time to sleep onset
Braam et al. (111)	*Dev Med Child Neurol*	Meta-analysis of placebo-controlled randomized trials of melatonin in individuals with intellectual disabilities and sleep problems. Nine studies were included. Various doses and formulations of melatonin were given. Melatonin decreased sleep latency by a mean of 34 min (*p* < 0.001), significantly decreased mean number of wakes per night (*p* = 0.024), and increased total sleep time by 50 min (*p* < 0.001). Specified reports on adverse effects were given in four studies. Adverse effects were minor and their incidence in both melatonin and placebo phases were the same. Patient groups in studies included in this meta-analysis were very heterogeneous
Guénolé et al. (112)	*Sleep Med Rev*	Systematic review of efficacy and safety of exogenous melatonin for treating disordered sleep in individuals with autism spectrum disorders: 4 case reports, 3 retrospective studies, 2 open-label clinical trials, 3 placebo-controlled trials. All studies supported the existence of a beneficial effect of melatonin on sleep in individuals with ASD with minor side effects. Limitations are: small sample, clinical heterogeneity of ASD and sleep disorders, varying methods used to measure sleep, confounding factors such as behavioral interventions and cross over design (no analysis of intention to treat). Melatonin doses ranged from 0.75 to 10 mg/day. The authors propose that future research on the efficacy of melatonin in children with ASD should include daytime functioning as a principal outcome measure. Only 6 patients of 205 presented side effects: daytime sleepiness, fogginess, dizziness, nocturnal enuresis, tiredness, headache, and diarrhea
Doyen et al. (113)	*Eur Child Adolesc Psychiatry*	Systematic review on pharmacokinetics data on melatonin and its role in sleep disorders and autism spectrum disorders. Authors reviewed 17 studies on effectiveness and side effects of melatonin in patients with AD, AS, PDDNOS, and Rett syndrome. Effectiveness on sleep disorders was found in all the studies, side effects were reported in 5 studies. Melatonin doses ranged from 0.5 to 10 mg. Melatonin seems to have anxiolytic properties. Most frequent reported side effects: infections, flu, epilepsy, intestinal disorders, and agitation
Rossignol and Frye (114)	*Dev Med Child Neurol*	Aim of the study: investigate melatonin-related findings in ASD including AD, AS, Rett syndrome, and PDDNOS. Eighteen studies on melatonin treatment on ASD patients were identified (5 RCT), 12 of them reported improvement in sleep with melatonin in 67% to 100% of the patients. Six studies reported improvement in daytime behavior (less behavioral rigidity, ease of management for parents and teachers, better social interaction, fewer temper tantrums, less irritability, more playfulness, better academic performance, and increased alertness). Melatonin doses ranged from 0.75 to 15 mg, age of patients ranged from 2 to 18 years, treatment duration ranged from 2 weeks to 4 years. Twelve studies explored side effects (headache, tiredness, dizziness, and diarrhea) in which 7 studies reported no side effects. Nine studies found low levels or abnormal circadian rhythm of melatonin in ASD. A correlation between these abnormal levels and autistic behaviors was found in 4 studies. Night time urinary excretion of melatonin metabolite (6-SM) was reported to be inversely correlated with the severity of impairments in verbal communication, play, and daytime sleepiness in patients with ASD. Five studies found genetic abnormalities of melatonin receptor and enzymes involved in melatonin synthesis
Reading (115)	*Child Care Health Dev*	Correlation between plasmatic levels of melatonin and autistic behaviors was found. Melatonin groups showed improvements in total sleep duration and sleep onset latency versus placebo groups but not on night awakenings
**LETTER TO THE EDITOR**		
Guénolé and Baleyte (116)	*Dev Med Child Neurol*	Response to the Rossignol and Frye review (73); Authors proposed that studies should separately explore sleep disorders in patients with ASD and sleep disorders in patients with Rett syndrome
Guénolé and Baleyte (117)	*Pediatr Neurol*	Response to the De Leersnyder et al. study (86) of open-label trial. The definition of «chronic sleep disorder»did not refer to international classifications. Half of the children manifested Smith-Magenis syndrome that involves specific abnormalities of melatonin secretion. Thus, results cannot apply to a population with ASD. The effects of melatonin should be studied separately in each neurodevelopmental disorder and with specific sleep diagnoses
**DISCUSSION/COMMENTARY**		
Jan and Freeman (92)	*Dev Med Child Neurol*	Discussion on melatonin use in children with ADHD, ASD, neurodevelopmental disabilities, epilepsy, and blindness. Exogenous melatonin seems to regulate endogenous melatonin secretion. It seems to be more effective in sleep–wake cycle disorders with sleep onset delay disorders. Night and morning awakenings seem to be more difficult to treat, such as sleep problems associated with cerebral lesions. The more the child shows mental or motor comorbidities, the more the melatonin dose is high
Lord (118)	*J Autism Dev Disord*	General brief discussion of melatonin and its potential for treating sleep problems in autism

As previously discussed (26), the treatment studies have a number of limitations. In some studies individuals covering a wide age range were included with pre-pubertal, pubertal, and post-pubertal individuals being studied (102, 103, 105). Reported differences in pineal melatonin secretion according to age and pubertal stage, coupled with the clearly developmental nature of autism, suggests that therapeutic effects of melatonin might vary considerably with age and pubertal status (23). Studies have also been limited by small sample sizes (90–93, 95, 96, 98, 102, 104). In larger studies, heterogeneous groups are often examined and have included blind individuals and individuals with various neurological disabilities with concomitant intellectual disability. In such studies, often results seen for the autism subgroup are not separately presented (97, 100, 103–107). The specificity and interpretation of the results with respect to autism are often unclear. Additional research is needed to determine whether and which findings might be specific to autism or whether melatonin’s effect might be similar across groups. It can be hypothesized that melatonin’s effects are due to actions on certain behavioral dimensions and that they can be observed across disorders. However, it should be pointed out that in our studies (18, 23), lower melatonin excretion was significantly associated with social communication impairments rather than with sleep problems. Future studies of melatonin in ASD should simultaneously examine melatonin levels, sleep problems, autistic behavioral impairments, and level of functioning so that a more complete picture can emerge.

It is worth noting that just a few of the therapeutic trials of melatonin have assessed effects on autistic behavioral impairments. These include reports of improved communication (105), reduced social withdrawal (82, 99), decreased stereotyped behaviors and rigidity (82, 102), and reduced anxiety (99, 103). Furthermore, at time the improvements noted were not sufficiently detailed. For example, Wright et al. (105) reported significant communication improvement without reporting separate verbal or non-verbal scores. In other instances, investigators did not use validated instruments; thus, Garstang and Wallis (102) reported improvements in rigidity based solely on parents’ and teachers’ comments, while Wasdell et al. (103) reported reductions in anxiety based on caregivers’ comments. All of the published clinical trials of melatonin in autism/ASD have used sleep measures as their main outcome variable and none have studied the dose–response relationship for melatonin. To reiterate, there is a real need for further studies on the therapeutic effects of melatonin that are conducted on large, relatively homogeneous samples and that employ validated behavioral assessments.

## Synchronization of Motor, Emotional, and Relational Rhythms: Therapeutic Benefits of the Early Start Denver Model in Autism Spectrum Disorder

Although there is a growing interest in the role of motor, emotional, and interpersonal rhythms in autism, there have been only a few attempts to focus on behavioral synchronization of social communication during therapeutic and educational intervention in autism. The ESDM, developed by Sally Rogers and Geraldine Dawson (119, 120), is a comprehensive behavioral and developmental early intervention approach designed for delivery to 12- to 60-month-old children. The model uses the knowledge of how typical infants, toddlers, and pre-schoolers develop, in addition to knowledge of the ways in which autism affects early development, in order to facilitate an appropriate developmental trajectory in young children with autism beginning at the earliest ages. The goals of the ESDM are to reduce the severity of disabling autism symptoms in very young children, and to accelerate children’s developmental rates in a multitude of domains, including cognitive, social–emotional, and language domains. In order to do so, there is a strong focus on interpersonal engagement marked by synchrony and reciprocity during teaching, as research demonstrates that these are closely tied to successful developmental outcomes (121–124). Within the ESDM, the therapist and child work together to develop and sustain coordinated, synchronous activity routines into which teaching opportunities are embedded. The ESDM reflects an understanding of the importance of synchrony of motor, emotional, and relational rhythms for the early development of young children with autism.

Thus, the ESDM allows one to initiate interaction with the child by synchronizing the therapist’s motor, emotional, and relational rhythms with the child’s rhythms (providing invariants/continuity). For example, if the child uses an object with a rhythmic motor pattern, the therapist will reproduce this behavior with exactly the same rhythmic pattern. The evolution of the child is then facilitated by introducing new and different rhythms (providing variants/discontinuity). In addition, the adjustment of the therapist’s behaviors, movements, level of arousal, and posture (for example, standing up, lying down, or sitting) to those of the child contributes to foundational invariants necessary during the initial phase. Furthermore, the ESDM requires the program to be implemented in different environments (such as home, school, and caregiver settings), which also contributes to the introduction of variants.

The ESDM is both a curriculum and a set of teaching practices. A specific developmental curriculum, administered every 3 months in typical practice, defines the skills to be taught at any given time. In addition, a manual of teaching practices outlines the ways in which these skills are to be taught. Embedded within both the curriculum and the set of teaching practices is a focus on rhythms and synchrony. In other words, when therapists are working within the model, a focus on rhythms permeates both what they teach and how they teach it.

The ESDM curriculum outlines skills to be taught in multiple areas, including cognitive, social–emotional, and language domains. The curriculum focuses heavily upon the teaching of skills that promote engagement with other people in a synchronous, rhythmic way. Many young children with autism enter an ESDM program with weaknesses in these skills, such as imitation, joint attention, orienting to name, and eye contact, and these have broad influences upon the manner in which these children can engage with others. It is well recognized that learning occurs within a social context and that social skills, such as imitation and shared attention, provide a foundation for many aspects of learning, including language, cognitive, and social–emotional abilities. Thus, the ESDM focuses heavily on the development of social engagement and interactional synchrony early on in order to further support learning of a wide range of skills.

One area of focus within the ESDM, which is closely tied to the concept of rhythmicity, is imitation. Young children with autism often exhibit deficits in their imitation skills, including those of imitating others’ facial expressions and movements, gestures, body actions, and actions on objects. From the beginning of a young child’s ESDM program, teaching of imitation is stressed, from basic imitation of actions on objects to more nuanced imitation of sound effects produced in play. Spontaneous and appropriate imitation of others is typically rhythmic and marked by mutual attention, continuous adaptation, and turn taking. The beginning stages of facilitating imitation and social engagement often start with imitating the child, thereby entering into a rhythmic interaction with the child. By imitating the child’s movements and establishing a synchronous interaction, eye contact and mutual engagement in the interaction are promoted (125).

Rhythmicity is also involved in the way teaching occurs within the ESDM upon multiple levels, from the basic structure of each interactive routine to the ways in which adults engage with children during interaction. Social communication is about sharing moments of synchrony with others, and the ESDM supports the emergence of interactional synchrony. The therapist facilitates the occurrence of synchronous moments and strings them together into routines into which the therapist can naturally embed teaching opportunities. The primary vehicle through which all teaching is accomplished in this model is the joint activity routine, which is permeated by moments of rhythmic, synchronous interaction, and enriched with positive affect. Adult and child are attuned to one another, both taking the lead, both following the other’s lead, taking turns, and creating a positive and motivating activity in conjunction with one another.

The joint activity routine, although flexible and naturalistic, adheres to a four-part structure: (1) opening, (2) theme, (3) elaboration, and (4) closing. This structure provides a set of invariants, against which multiple variants can occur at differing levels. In order to begin engagement within a joint activity routine, the adult works hard to *find the child’s smile*. This can often be accomplished by getting into the child’s own rhythm (imitating a child’s actions is a very powerful tool to facilitate motivated response and interaction) or by finding a rhythm that the child likes. For example, perhaps a child finds a toy drum and begins banging on it with his hand. In the ESDM, the therapist would join the child and would likely take her/his own drum and imitate the child’s actions, joining his rhythm. This would be considered the opening phase of the joint activity routine.

Next, the child and adult would develop a theme. This can be considered a continuation of the rhythm introduced during the opening phase – in our example, banging a drum slowly with hands. The adult and child would take turns doing so, each leading and each following. The skillful adult would embed teaching opportunities into these turns – perhaps a focus on eye contact during dyadic engagement, or on imitation of actions on objects, or on giving and taking objects with eye contact. Both partners then play within this rhythm for a while, until one introduces a variant, or elaboration. In the ESDM, elaboration occurs by introducing a change into the joint activity routine, a change in the rhythm which often allows different teaching targets to be practiced. In this example, the child and adult might start to play peek-a-boo behind the drums. A new rhythm would need to be established, and both partners would work together in order to do so. An elaboration of this sort is a rhythmic fluctuation occurring against a background of invariants (e.g., the same interactive partner, the same material, the same setting), which allows the child to learn to adapt to change and teaches the child how to engage with people and materials differently. Finally, this rhythm would likely begin to fade, the teaching value of the activity would start to diminish, and/or the child would begin to lose motivation. The fourth stage of the joint activity routine – the closing – is the last part of the joint activity routine. Adult and child might help one another clean up the materials, and then both partners would begin an entirely new joint activity routine. This could often involve a change in location, activity level, or teaching domain, and an entirely new set of coordinated interactions would be developed.

Within the joint activity routine, synchrony pervades within several different levels and in several different areas. Adult and child are attuned to one another and responsive to one another in terms of sensory input and output, motor actions, and emotions. Coordinated dyadic engagement, well-balanced in terms of leader and follower, pervades a high-quality joint activity routine. It is quite natural to view this ever-present synchrony in terms of a focus on shared nuanced rhythms.

There is also a focus on rhythmicity within some of the broader teaching practices of the ESDM; for example, in the way in which a teaching session is constructed. As mentioned above, teaching is delivered within joint activity routines. For a therapist-delivered session, these routines are strung together within a session to occupy a full 1- or 2-h time period. Within that time period, therapist and child move around to occupy several spaces. In a well-coordinated way, they engage together in a joint activity routine at the table, and then may move onto the floor for the next. They may sit together, then they may stand or walk or run. The child’s level of arousal is carefully monitored, and the session is marked by alternations of quiet thoughtfulness and active play, all coordinated artfully by a skilled adult. All of these changes occur against a stable background, as the session is marked by stability and continuity, with the introduction of variants when appropriate. The ESDM, a comprehensive, behavioral, and developmental early intervention designed for infants and toddlers with autism, is rooted within a sense of and appreciation for rhythmicity and synchrony at multiple levels, ranging from the specific skills that are taught to how those skills are taught, woven together and supported by a broader structure focused on maintaining a well-coordinated, synchronous set of dyadic interactions.

## Conclusion: Toward an Integrative Approach Combining the Use of Melatonin with the ESDM

Taken all together, ASD could be seen as a disorder of rhythmicity with, more specifically, impairments in the synchrony of rhythms. Alternatively, such asynchrony might play an important role in a possibly large subgroup of individuals that forms part of the heterogeneous ASD category. In this article, we proposed an integrative approach to study desynchronization in biological and psychological rhythms in ASD and develop an etiopathogenic hypothesis as well as therapeutic perspectives for ASD based on this integrative approach. Indeed, this integrated physiological and psychological approach opens important therapeutic perspectives for ASD based on regulation of physiological rhythms (in particular, through the use of chronobiotics such as melatonin, and also through light exposure, use of regularly scheduled bedtime, wake up, meals, or activities) combined with synchronization of motor, emotional, and relational rhythms through developmental behavioral intervention such as the ESDM. Further studies are required to better ascertain the underlying mechanisms of physiological alterations induced by temporal desynchronization and to better understand the role of biological rhythms and rhythmicity in the development of social communication, repetitive behaviors, and interests or adaptation to changes, and therefore in the development of autism involving impairments in these domains.

## Conflict of Interest Statement

The authors declare that the research was conducted in the absence of any commercial or financial relationships that could be construed as a potential conflict of interest.
